# ﻿A comparison of gene organisations and phylogenetic relationships of all 22 squamate species listed in South Korea using complete mitochondrial DNA

**DOI:** 10.3897/zookeys.1129.82981

**Published:** 2022-11-10

**Authors:** Daesik Park, Il-Hun Kim, Il-Kook Park, Alejandro Grajal-Puche, Jaejin Park

**Affiliations:** 1 Kangwon National University, Chuncheon, Republic of Korea Kangwon National University Chuncheon Republic of Korea; 2 National Marine Biodiversity Institute of Korea, Seochun, Republic of Korea National Marine Biodiversity Institute of Korea Seochun Republic of Korea; 3 Northern Arizona University, Flagstaff, Arizona, USA Northern Arizona University Flagstaff United States of America

**Keywords:** Full mitochondrial genome, Korea, phylogeny, rearrangement, Squamata, *tRNA-Pro*, *WANCY*

## Abstract

Studies using complete mitochondrial genome data have the potential to increase our understanding on gene organisations and evolutionary species relationships. In this study, we compared complete mitochondrial genomes between all 22 squamate species listed in South Korea. In addition, we constructed Maximum Parsimony (MP), Maximum Likelihood (ML) and Bayesian Inference (BI) phylogenetic trees using 13 mitochondrial protein-coding genes. The mitochondrial genes for all six species in the suborder Sauria followed the same organisation as the sequenced Testudines (turtle) outgroup. In contrast, 16 snake species in the suborder Serpentes contained some gene organisational variations. For example, all snake species contained a second control region (*CR2*), while three species in the family Viperidae had a translocated *tRNA-Pro* gene region. In addition, the snake species, *Elapheschrenckii*, carried a *tRNA-Pro* pseudogene. We were also able to identify a translocation of a *tRNA-Asn* gene within the five tRNA (*WANCY* gene region) gene clusters for two true sea snake species in the subfamily Hydrophiinae. Our BI phylogenetic tree was also well fitted against currently known Korean squamate phylogenetic trees, where each family and genus unit forms monophyletic clades and the suborder Sauria is paraphyletic to the suborder Serpentes. Our results may form the basis for future northeast Asian squamate phylogenetic studies.

## ﻿Introduction

Elucidating comparative gene organisations and phylogenetic relationships between species is essential for improving our understanding of their evolutionary histories and may allow for the successful conservation of endangered taxa (Vane-Wright et al. 1991; Crozier 1992; Boore et al. 1995; Mouquet et al. 2012). Knowledge of genetic organisational variations often give further insights into the evolutionary history of species and could be helpful in developing conservation management plans (Swenson 2019). Additionally, gene organisation data provide reliable phylogenetic information within the evolutionary branches (Boore 1999). Furthermore, defining a phylogenetic species, using mitochondrial and nuclear genes, is often vital to designate if a species is endangered at a national level (An et al. 2010).

The mitochondrial genome is an important model system used to understand genome structure, molecular evolution and phylogenetic relationships amongst vertebrates (Moritz and Brown 1986; Pääbo et al. 1991; Inoue et al. 2003; Krishnan et al. 2004; Jiang et al. 2007; Qian et al. 2018). Specific mitochondrial genes, such as the 12S ribosomal RNA (*12S rRNA*), 16S ribosomal RNA (*16S rRNA*), NADH dehydrogenase subunit 4 (*ND4*), cytochrome c oxidase subunit I (*COI*) and cytochrome b (*Cytb*) have been often used to compare phylogenetic relationships between vertebrates (Malhotra and Thorpe 2004; Rivera et al. 2018; Sidharthan et al. 2021). Although phylogenetic studies using mitochondrial sequences have both advantages and disadvantages (Rubinoff and Holland 2005), using the complete mitochondrial genome may provide higher phylogenetic resolution between species (Inoue et al. 2003; Nardi et al. 2003; Qian et al. 2018). In addition, a comparison of gene organisations, using complete mitochondrial genomes, could give a more precise understanding of the evolutionary histories between species (Boore 1999). To date, a total of 6,781 complete vertebrate mitochondrial genome sequences have been deposited in GenBank (National Center for Biotechnology Information; NCBI), of which 303 are squamates. These 303 sequenced mitochondrial genomes account for approximately 3.8% of the total 7,953 known squamate species (http://www.reptile-database.org/).

Squamates are the most speciose order amongst reptiles, have high ecological and morphological diversity and are found nearly worldwide, with the exception of Antarctica (Greene 1997; Pianka et al. 2003; Vitt and Caldwell 2013). Squamates are divided into two suborders, Sauria and Serpentes. In South Korea, there are six saurian species (five lizards and one gecko species) across four genera and 16 snake species across 10 genera. Together, these squamate species account for 71.0% of all South Korean reptiles (two orders, 11 families, 22 genera and 31 species). In South Korea, there have been several phylogenetic studies mapping reptilian phylogenetic associations. Lee (2010) morphologically demarcated several lizard species, while Lee (2011) combined two *Elapheschrenckii* subspecies by comparing specific mitochondrial genes and microsatellite alleles. *Rhabdophistigrinus*, listed in both South Korea and Japan, was recently reclassified as *R.lateralis*, based on mitochondrial genes (Takeuchi et al. 2014). More recently, Kim (2018) applied morphological data in addition to complete mitochondrial genome sequences to classify sea snake species found in Korean waters. Only one large-scale reptilian phylogenetic study has phylogenetically distinguished between Korean serpent species, based on both morphological and isozyme data (Paik 1982). Thus far, there have been no phylogenetic reconstructions using gene organisations amongst Korean squamates using complete mitochondrial genomes.

Recent phylogenetic molecular advances have made it more possible to compare complete mitochondrial genome relationships between species (Lee et al. 2021; Zhang et al. 2021; Vanerelli et al. 2022). These molecular advances make it a prime occasion to update our understanding of the phylogenetic relationships amongst squamates, specifically in South Korea. In this study, we compared mitochondrial gene organisations and constructed three types of phylogenetic trees amongst all 22 squamate species listed in South Korea, using complete mitochondrial sequence data. These results have the potential to impact future squamate phylogenetic studies in northeast Asia.

## ﻿Materials and methods

Complete mitochondrial DNA (mtDNA) sequences, for all 22 squamate species listed in South Korea, were downloaded from GenBank (NCBI). We tried to obtain mtDNA sequence data from individuals, captured within South Korea. When it was not possible, we used mtDNA data from specimens collected in China, Japan or Russia (Table 1) that were within the distribution range of the same species listed in South Korea (Takeuchi et al. 2014; Lee et al. 2022). The downloaded, foreign-originated sequences from GenBank showed high similarity with the preliminary BLAST analysis using partial 16S ribosomal RNA (*16S rRNA*), cytochrome oxidase subunit I (*COI*) or cytochrome b (*Cytb*) sequences of South Korean specimens (Jeong et al. 2013). For *Hydrophiscyanocinctus* and *H.melanocephalus*, we used the data from individuals caught at locations nearest to South Korea, as there were no data available from South Korean specimens. After confirming complete mitochondrial genes for each species, we arranged and compared the mitochondrial genes between species, based on transfer RNA (tRNA) positions and ribosomal RNA (rRNA) and protein-coding gene (PCG) positions, which were determined using tRNAscan-SE 2.0 (Chan and Lowe 2019) and MITOS Webserver (Bernt et al. 2013), respectively. We excluded *H.cyanocinctus* from the analysis due to the uncertainty of the nucleotide sequence in non-protein-coding regions, such as rRNA, tRNA, O_L_ and CR within a cluster of five tRNA genes (*WANCY* gene region).

**Table 1.** All 22 analysed squamate species listed in South Korea and their sampling locations analysed in this study. Original sample location, GenBank accession number and mt-genome base pair length are provided.

Suborder	Family	Species	Location	GenBank No.	Length (bp)
Sauria	Gekkonidae	*Gekkojaponicus*	S. Korea	KR996131	16,544
	Scincidae	*Scincellavandenburghi*	S. Korea	KU646826	17,103
		*Scincellahuanrenensis*	S. Korea	KU507306	17,212
	Lacertidae	*Takydromusamurensis*	China	KU641018	17,333
		*Takydromuswolteri*	China	JX181764	18,236
		*Eremiasargus*	S. Korea	JQ086345	18,521
Serpentes	Colubridae	*Elaphedione*	Russia	MH460961	17,172
		*Elapheschrenckii*	China	KP888955	17,165
		*Oocatochusrufodorsatus*	China	KC990020	17,159
		*Rhabdophislateralis*	China	KU641019	17,415
		*Hebiusvibakari*	China	KP684155	17,259
		*Lycodonrufozonatus*	China	KJ179950	17,188
		*Orientocoluberspinalis*	S. Korea	MT304473	17,196
		*Sibynophischinensis*	S. Korea	KF360246	17,163
	Viperidae	*Gloydiusussuriensis*	China	KP262412	17,208
		*Gloydiusbrevicaudus*	China	EU913477	17,227
		*Gloydiussaxatilis*	S. Korea	MW143075	17,223
	Elapidae	*Hydrophiscyanocinctus*	China	MK953550	17,750
		*Hydrophismelanocephalus*	Japan	MK775532	17,182
		*Hydrophisplaturus*	S. Korea	MK775530	18,101
		*Laticaudalaticaudata*	S. Korea	KY496323	17,209
		*Laticaudasemifasciata*	S. Korea	KY496325	17,170
Cryptodira	Cheloniidae	*Cheloniamydas*	Cyprus	JX454990	16,495
		*Carettacaretta*	USA	JX454983	16,454

To construct the phylogenetic tree of all 22 squamate species listed in South Korea, we used 13 mitochondrial PCGs (Fenn et al. 2008; Douglas and Gower 2010; He et al. 2010). The sequence of each PCG was extracted and aligned using MUSCLE, with a maximum of 20 iterations (Edgar 2004). Aligned sequence genes were concatenated into one sequence using Geneious v.9.1.8 (Kearse et al. 2012). We used two sea turtle species, *Carettacaretta* and *Cheloniamydas*, as outgroups for our constructed phylogenetic tree. We used PAUP 4.0a168 (Swofford 2001) to create the Maximum Parsimony (MP) phylogenetic tree. To increase the reliability of the MP phylogenetic tree, we performed 1,000 bootstrap iterations. For estimating the MP tree, we applied a heuristic search using tree bisection and reconnection (TBR) and branch swapping approaches. We used RAxML v.7.2.8 (Stamatakis 2006) to create the Maximum Likelihood (ML) phylogenetic tree. To select the best nucleotide model for the ML analysis, we used jModelTest 2.1.10 (Darriba et al. 2012) and selected the GTR GAMMA I model through AIC calculations. The rapid bootstrapping value was calculated while selecting the best-scoring tree from 100 ML trees. To construct the Bayesian Inference (BI) phylogenetic tree, we used MrBayes v.3.2.6 (Huelsenbeck and Ronquist 2001). For the BI phylogenetic tree, each substitution model and rate variation was set to GTR and gamma, respectively. For the Markov Chain Monte Carlo (MCMC) analysis of the BI tree, the chain length was set to 1,000,000, while the subsampling frequency was set to 100. The MCMC heated chain was also set to 4, the heated chain temperature was set to 0.2 and the burn-in cut-off was at 50,000.

## ﻿Results

**Figure 1.**
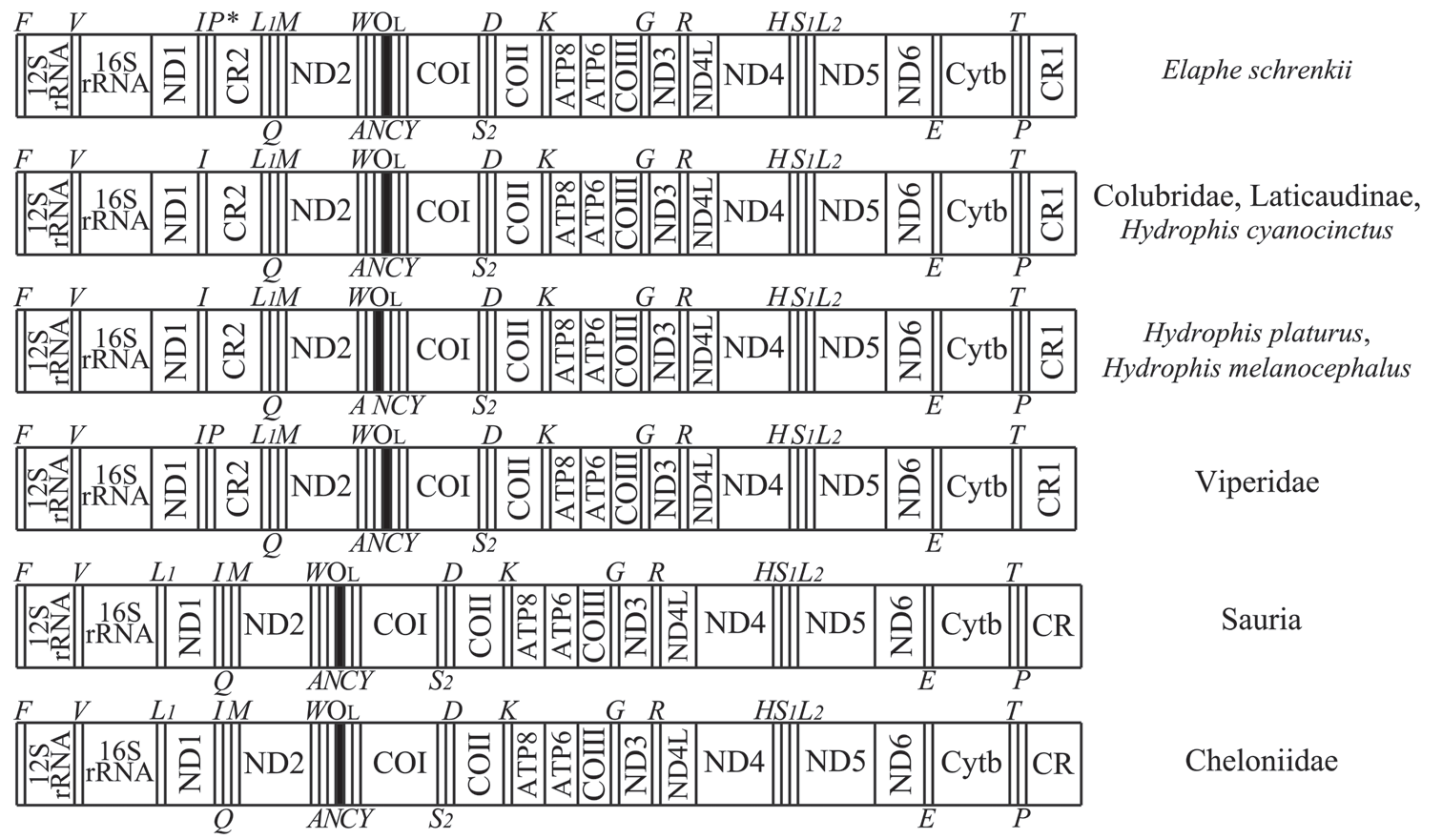
Comparison of the mitochondrial gene organisation for all 22 squamate species listed in South Korea. Two sea turtle species (*Carettacaretta* and *Cheloniamydas*), served as outgroups. *L1*, *L2*, *S1*, *S2*. P* represent the following genes: *tRNA-Leu* (UUR), *tRNA-Leu* (CUN), *tRNA-Ser* (AGY), *tRNA-Ser* (UCN) and a pseudo *tRNA-Pro* gene, respectively. tRNA genes are abbreviated by the corresponding one-letter amino acid codes, such as *F*, *V*, *I*, *M* etc.

All six species within the suborder Sauria matched the mitochondrial organisation of the sea turtle outgroup (Fig. 1). In contrast, all 16 snake species in the suborder Serpentes had a second control region (*CR2*) between the NADH dehydrogenase subunit 1 (*ND1*) and the NADH dehydrogenase subunit 2 (*ND2*). These 16 snake species also had translocated *tRNA-Leu* (UUA) genes between *tRNA-Ile* and *tRNA-Gln*. In addition, three species in the family Viperidae had a translocated *tRNA-Pro* gene. The *tRNA-Pro* gene was originally between *tRNA-Thr* and *CR1* genes, but was translocated in between the *tRNA-Ile* and *CR2* genes. Moreover, the three viperid species lacked any *tRNA-Pro* pseudogenes observed in the other serpent species. In *H.platurus* and *H.melanocephalus*, the *tRNA-Asn* gene, originally located between *tRNA-Ala* and the light strand replication origin (O_L_) in other snakes, had also been translocated between the O_L_ region and the *tRNA-Cys* gene within the WANCY cluster region. Finally, a pseudo-*tRNA-Pro* gene was discovered between the *tRNA-Ile* and *CR2* genes in *Elapheschrenckii*.

**Figure 2.**
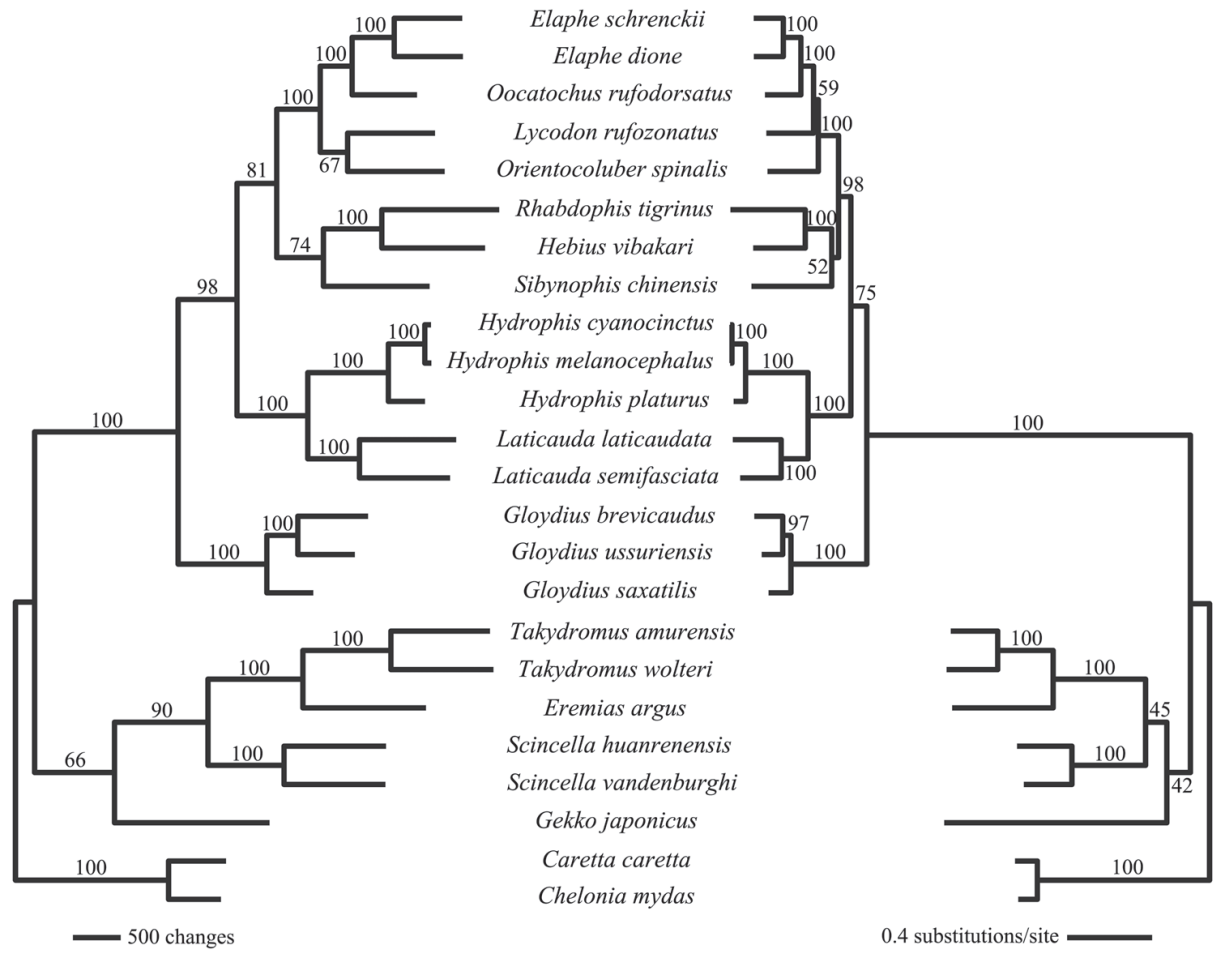
Maximum Parsimony (MP) tree (left) and Maximum Likelihood (ML) tree (right) for all 22 squamates listed in South Korea. Both MP and ML trees are based on 13 mitochondrial protein-coding genes. *Carettacaretta* and *Cheloniamydas* are outgroup species. Bootstrap values are denoted on each tree branch.

**Figure 3.**
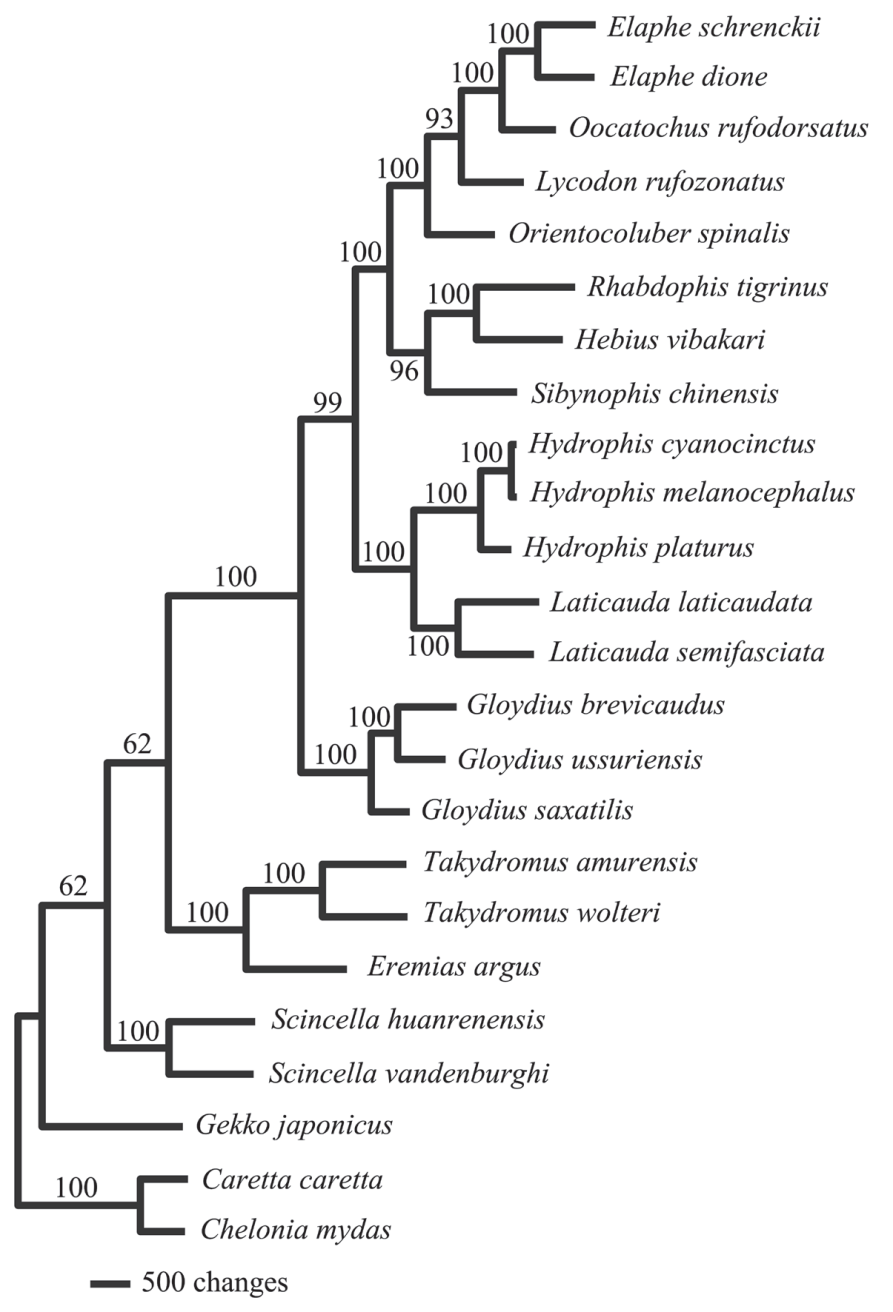
The constructed Bayesian Inference (BI) tree for all 22 squamate species listed in South Korea, based on 13 mitochondrial protein-coding genes. Two cheloniid species (*Carettacaretta* and *Cheloniamydas*) were used as outgroups. Bayesian posterior probabilities are denoted on each tree branch.

The aligned sequence data for the 13 mitochondrial protein-coding genes were concatenated to 11,581 bp. The suborders Sauria and Serpentes each formed monophyletic clades in both MP and ML phylogenetic trees (Fig. 2). In contrast, all six species within the suborder Sauria were paraphyletic to the suborder Serpentes in the BI tree (Fig. 3). Branching patterns were the same between Viperidae and Elapidae families amongst our three constructed phylogenetic trees. Specifically, two species in the family Viperidae, *Gloydiusussuriensis* and *G.brevicaudus*, were more closely related, while *G.saxatilis* formed a sister clade. Within the genus *Hydrophis*, *H.cyanocinctus* and *H.melanocephalus* clustered more closely together than *H.platurus*. For the family Colubridae, species in the BI tree were paraphyletic, which differed from our constructed ML and MP trees. In the BI tree, *Gekkojaponicus* was basal to all other saurian species.

## ﻿Discussion

We compared gene organisations and elucidated phylogenetic relationships, using complete mitochondrial genomes, for all 22 squamate species listed in South Korea. Using this complete mtDNA approach, we were able to uncover unique evolutionary insights amongst South Korean squamates.

Relative to the six saurian species, we discovered that all 16 snake species in the suborder Serpentes had an additional control region (*CR2*) and a translocated *tRNA-Leu* (UUR) gene. These genetic characteristics have been documented in many other snake species, except for the infraorder Scolecophidia (Douglas et al. 2006; Jiang et al. 2007). The *CR2* was located between *ND1* and *ND2* genes and may be an example of a non-independent replication event (Dover 1982; Kumazawa et al. 1998; Dong and Kumazawa 2005). The presence of two control regions may increase the rate of mitochondrial gene replication (Jiang et al. 2007). Duplication of the *CR* is not novel amongst vertebrates and has been identified in fishes (Lee et al. 2001; Inoue et al. 2003), frogs (Sano et al. 2005; Huang et al. 2019), lizards (Kumazawa and Endo 2004; Amer and Kumazawa 2005) and birds (Eberhard et al. 2001; Sammler et al. 2011). The translocation of *tRNA-Leu* (UUR) gene, identified in this study, is considered an ancestral trait of the infraorder Alethinophidia (Dong and Kumazawa 2005). In vertebrates, the *tRNA-Leu* gene has been shown to alter the transcribed ratios between the rRNA and protein-coding gene as terminators for heavy-stranded transcripts (Fernández-Silva et al. 2003). The presence of the *CR2* and the translocation of the *tRNA-Leu* gene might also prevent a thermodynamic depression of transcriptional enzymes in snakes (Jiang et al. 2007).

All three *Gloydius* species, listed in South Korea, had translocated *tRNA-Pro* gene regions, which is consistent with other previously reported Viperidae species, such as *Agkistrodonpiscivorus* (Jiang et al. 2007), *Caususdefilippi* (Castoe et al. 2009), *Bothropspubescens* (GenBank No. MG182598) and *Viperaberus* (Gao et al. 2018). In contrast, we did not detect any half-sized *tRNA-Pro* pseudogenes from the three analysed *Gloydius* species, although the half-sized pseudogenes have been previously reported in a closely-related species, *G.strauchi* (GenBank No. MF523224). The half-sized tRNA-Pro pseudogene, adjacent to the 5’ end of *CR1*, has also been reported in *Ovophisokinavensis* (Kumazawa et al. 1996) and in several other crotaline species, such as *Protobothropskaulbacki* (Kang et al. 2017), *Trimeresurusalbolabris* (Song et al. 2015) and *T.sichuanensis* (Zhu et al. 2016). The half-sized *tRNA-Pro* pseudogene is considered a shared ancestral trait amongst crotaline species and may have been lost and subsequently recovered independently between species (Wüster et al. 2008).

**Figure 4.**
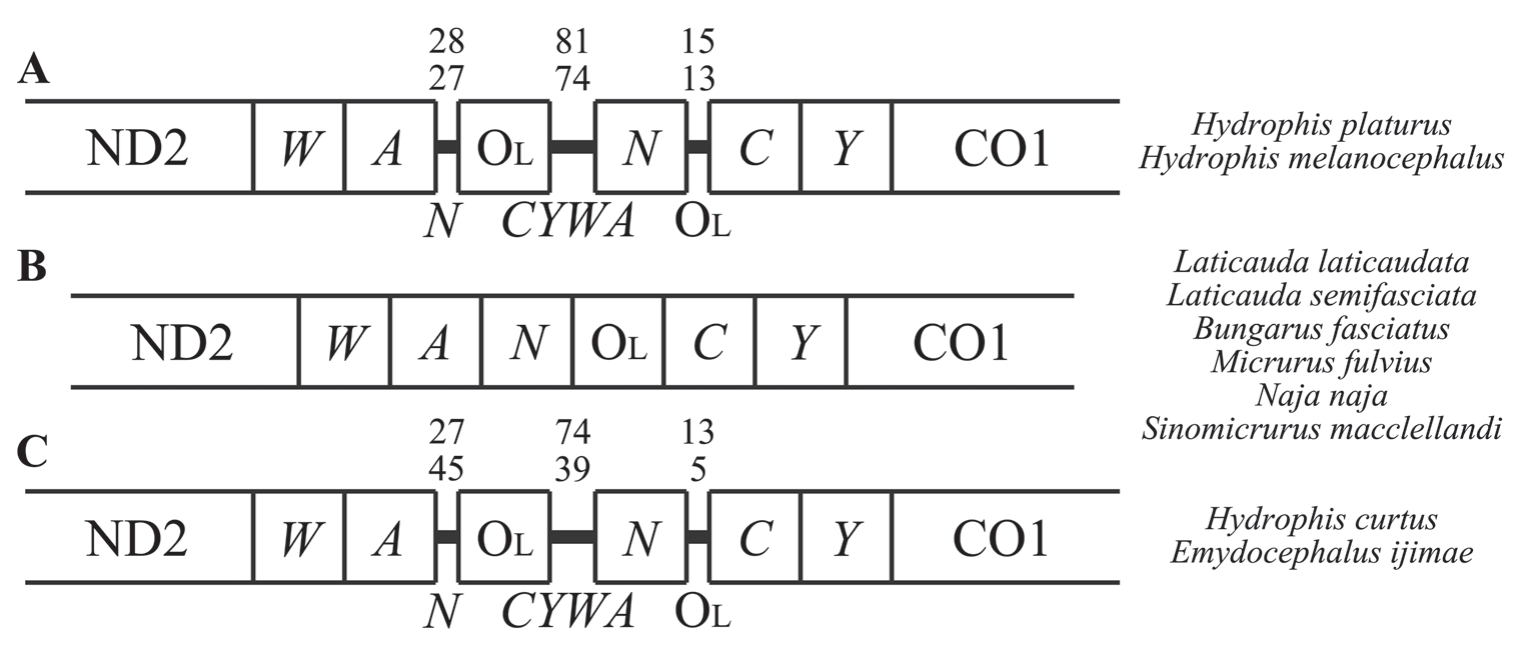
Intergenic spacers located in the cluster of five tRNA genes (*WANCY* gene region) of two sea snake species (*Hydrophisplaturus* and *H.melanocephalus*) found off South Korean coastal waters **A** intergenic spacers located in the *WANCY* gene region of two sea krait species (*Laticaudalaticaudata* and *L.semifasciata*), four terrestrial cobras (*Bungarusfasciatus*, *Micrurusfulvius*, *Najanaja* and *Sinomicrurusmacclellandi*) **B** and two true sea snake species downloaded from GenBank (*H.curtus* and *Emydocephalusijimae*) **C** intergenic spacer lengths amongst Hydrophiinae species are above the intergenic spacer in the same order as the species are listed. The hypothesised tRNA genes lost due to random deletions are written below the intergenic spacers. The tRNA genes are abbreviated by the corresponding one-letter amino acid codes, such as *W*, *A*, *N* etc.

In *H.platurus* and *H.melanocephalus*, the *tRNA-Asn* gene was translocated between the O_L_ and *tRNA-Cys* genes. The *tRNA-Asn* gene is usually located between *tRNA-Ala* and O_L_ in the WANCY region in other snakes and vertebrates (Boore 2000; Qian et al. 2018; Fig. 4). To our knowledge, this is the first finding of a *tRNA-Asn* translocation in the infraorder Alethinophidia. Mitochondrial genetic rearrangements are often found near control or WANCY regions in serpents (Qian et al. 2018). Tandem duplication random loss (TDRL) models often explain that some gene rearrangements may occur due to random paralog deletions (Moritz et al. 1987; Boore 2000; Xiaokaiti et al. 2022). These random paralog deletions may explain our results, as paralog deletions have caused gene rearrangements in other vertebrates including lizards, marsupials, birds and fishes (Moritz and Brown 1986, 1987; Moritz et al. 1987; Pääbo et al. 1991; Boore 2000; Inoue et al. 2003). We were also able to identify a novel gene rearrangement in sea snakes found off the coasts of South Korea. To our knowledge, this gene rearrangement has not been detected in other elapid species, including cobras (Yan et al. 2008; Castoe et al. 2009; Singchat et al. 2019) and sea kraits (Kim et al. 2018).

The existence of the intergenic spacers, which are non-coding regions between genes, is evidence of certain genetic deletions (San Mauro et al. 2006; Xiaokaiti et al. 2022). The gene rearrangement observed in the true sea snake species may be the result of a duplication of the *WANCY* genes and subsequent deletions of certain parts of other genes (Fig. 4A). We were also able to corroborate the occurrence of similar gene rearrangements in two other foreign sea snake species, *Emydocephalusijimae* and *H.curtus*, for which complete mt-genome were available (GenBank MK775531, MT712129; Yi et al. 2019; Zhang and Yan 2020) (Fig. 4B, C). This WANCY gene rearrangement was not present in the four sequenced terrestrial cobra species (*Bungarusfasciatus*, *Micrurusfulvius*, *Najanaja* and *Sinomicrurusmacclellandi*; GenBank No. EU579523, GU045453, DQ343648, MT547176; Yan et al. 2008; Castoe et al. 2009; Yao et al. 2020). Uploading and comparing additional mt-genome data for closely-related Australian terrestrial cobras and sea snakes may give further insights into elapid speciation and possibly adaptation from terrestrial to oceanic ecosystems.

Our constructed BI phylogenetic tree was well fitted amongst known Korean squamate phylogenies. In our BI tree, the suborder Sauria was paraphyletic to the superfamily Lacertoidea and was sister to the suborder Serpentes. In addition, the family Gekkonidae formed an independent branch at the most basal position, which is consistent with previous studies (Townsend et al. 2004; Vidal and Hedges 2005; Hugall et al. 2007; Wiens et al. 2012; Pyron et al. 2013; Reeder et al. 2015). Our constructed BI tree also corroborated a recent phylogenetic investigation which used both morphological characters and specific mitochondrial and nuclear genes of 200 squamate species (Reeder et al. 2015). Due to the parallels between our constructed BI tree and the aforementioned study, we hypothesise that South Korean squamates most likely speciated in this manner.

In this study, we used complete mitochondrial sequenced genomes to determine mitochondrial gene organisations and phylogenetic relationships amongst all 22 squamate species listed in South Korea. The analysed species appear to have several unique mitochondrial rearrangements, including family and order-specific gene duplications and translocations. Overall, our constructed BI phylogenetic tree was well fitted amongst Korean squamates and is consistent with other phylogenetic studies which utilised specific mt-gene sequences. These results may form the basis of future phylogenetic investigations, clarifying northeast Asian squamate speciation.
